# Co-distribution and co-infection of chikungunya and dengue viruses

**DOI:** 10.1186/s12879-016-1417-2

**Published:** 2016-03-03

**Authors:** Luis Furuya-Kanamori, Shaohong Liang, Gabriel Milinovich, Ricardo J. Soares Magalhaes, Archie C. A. Clements, Wenbiao Hu, Patricia Brasil, Francesca D. Frentiu, Rebecca Dunning, Laith Yakob

**Affiliations:** Research School of Population Health, Australian National University, Acton, ACT 2601 Australia; Environmental Health Institute, National Environment Agency, Singapore, 138667 Singapore; School of Public Health and Social Work, Queensland University of Technology, Kelvin Grove, QLD 4059 Australia; School of Veterinary Science, University of Queensland, Gatton, QLD 4343 Australia; UQ Children’s Health Research Centre, University of Queensland, South Brisbane, QLD 4101 Australia; Instituto Nacional de Infectologia Evandro Chagas/ Fiocruz, Rio de Janeiro, Brazil; School of Biomedical Sciences and Institute for Health and Biomedical Innovation, Queensland University of Technology, Kelvin Grove, QLD 4059 Australia; Formerly School of Biomedical Sciences, University of Queensland, St Lucia, QLD 4072 Australia; Department of Disease Control, London School of Hygiene and Tropical Medicine, London, WC1E 7HT UK

**Keywords:** Chikungunya, Dengue, Virus, Coinfection, Review

## Abstract

**Background:**

Chikungunya and dengue infections are spatio-temporally related. The current review aims to determine the geographic limits of chikungunya, dengue and the principal mosquito vectors for both viruses and to synthesise current epidemiological understanding of their co-distribution.

**Methods:**

Three biomedical databases (PubMed, Scopus and Web of Science) were searched from their inception until May 2015 for studies that reported concurrent detection of chikungunya and dengue viruses in the same patient. Additionally, data from WHO, CDC and Healthmap alerts were extracted to create up-to-date global distribution maps for both dengue and chikungunya.

**Results:**

Evidence for chikungunya-dengue co-infection has been found in Angola, Gabon, India, Madagascar, Malaysia, Myanmar, Nigeria, Saint Martin, Singapore, Sri Lanka, Tanzania, Thailand and Yemen; these constitute only 13 out of the 98 countries/territories where both chikungunya and dengue epidemic/endemic transmission have been reported.

**Conclusions:**

Understanding the true extent of chikungunya-dengue co-infection is hampered by current diagnosis largely based on their similar symptoms. Heightened awareness of chikungunya among the public and public health practitioners in the advent of the ongoing outbreak in the Americas can be expected to improve diagnostic rigour. Maps generated from the newly compiled lists of the geographic distribution of both pathogens and vectors represent the current geographical limits of chikungunya and dengue, as well as the countries/territories at risk of future incursion by both viruses. These describe regions of co-endemicity in which lab-based diagnosis of suspected cases is of higher priority.

**Electronic supplementary material:**

The online version of this article (doi:10.1186/s12879-016-1417-2) contains supplementary material, which is available to authorized users.

## Background

Dengue is the most important arbovirus in global public health [1]. It is spread by the bite of the highly anthropophilic *Aedes aegypti* mosquito, and to a lesser extent, by *Ae. albopictus*. Over half of the world’s population inhabit areas at risk of dengue infection [2, 3]. Currently, the WHO reports its presence in more than 125 countries [4] and recent modelling suggest as many as 390 million infections occur annually [5]. Dengue fever results from infection with any of the four closely related dengue serotypes: DENV-1, -2, -3 and -4. In a minority of cases, infection can progress to life-threatening condition such as dengue haemorrhagic fever (DHF). Infection confers protection from subsequent exposure to the same serotype but does not protect against the others [6], and sequential infections from other serotypes increases the risk of DHF [7]. Case fatality rates of dengue infection vary between 0.5 % – 3.5 % [8, 9].

Chikungunya virus (CHIKV) is an alphavirus also transmitted by *Aedes* spp. mosquitoes. There are three distinct evolutionary clades: West African, Central/East African and Asian CHIKV [10]. Historically, chikungunya was not considered a life-threatening infection but recent epidemiological evidence suggests a case fatality rate of around 0.1 % (mostly affecting the elderly) [11]. A variant of CHIKV first detected in a 2004 Kenyan outbreak spread globally through international travel, leading to autochthonous transmission events in islands of the Indian Ocean in 2005/6, India in 2005/6 and Europe in 2007 [12, 13]. This rapid spread of chikungunya demonstrated for the first time both the devastating magnitude of modern-day outbreaks (India was the worst affected country with over 1.4 million infections) and the ability of transmission in temperate regions [14–16]. More recently, in 2013, the first case of locally transmitted case of CHIKV outside Africa, Asia and Europe was reported in French Guyana; since then, 44 countries in the Americas have reported chikungunya cases in their territories [17].

Both pathogens are transmitted by the same *Aedes* spp. mosquitoes and so there is a reasonable expectation that the epidemiology of chikungunya and dengue infections is temporally and spatially related. Moreover, because symptoms presented by infected patients are similar and diagnosis of both infections is predominantly symptom-based, there will inevitably be ambiguity in disease recognition in inhabitants of endemic/epidemic regions and returning travellers. Therefore, the aims of this study were to: 1) determine the geographic limits of chikungunya, dengue and the principal mosquito vectors of both viruses, 2) review the available evidence of chikungunya and dengue co-infections, and 3) describe the clinical significance of chikungunya and dengue co-infection.

## Methods

### Search strategy for chikungunya and dengue co-infection

A search was conducted in three medical and life sciences databases (PubMed, Scopus and Web of Science) from their inception until May 2015 for all relevant articles. The search terms included were co-infection and concurrent isolation along with chikungunya, dengue and breakbone fever. The specific keywords and connectors used in the search strategy for each database are listed in S1. Review of bibliographies of papers was also carried out to ensure completeness of inclusion of all relevant studies.

### Study selection for chikungunya and dengue co-infection

Studies eligible for inclusion were those describing detection of both viruses in the same patient. Studies describing virus detection either through direct methods (including qPCR) or indirect methods (e.g., immunoglobulin M and IgG detection with ELISA) were included. Papers were excluded if they did not report the number of co-infected patients; if clinical diagnosis of dengue and chikungunya was not confirmed by laboratory tests; or if data were presented in a non-extractable format (S2).

Two authors (LFK and SL) independently examined all the citations by title and abstracts for studies that met the inclusion criteria. Full-text version articles of all potentially relevant studies were retrieved and independently extracted. Extracted data were cross-checked by the same two authors, discrepancies during the selection of studies or data extraction were resolved through discussion and consensus following independent evaluation by another author (GM). The extracted data included study characteristics (design, location and year) and data regarding the infection (laboratory method used for DENV/CHIKV detection, number of cases, isolated strains of DENV/CHIKV and vector responsible for the transmission).

### Mapping the distribution of Ae. aegypti and Ae. albopictus and the occurrence of chikungunya, dengue and co-infection cases

To synthesise current understanding of chikungunya-dengue co-distribution, we collated global distribution data for both pathogens as well as for both *Ae. aegypti* and *Ae. albopictus*. By combining data from WHO, CDC, peer-reviewed literature and Healthmap alerts, we created up-to-date global distribution maps for both dengue and chikungunya. This exercise was greatly facilitated in the case of dengue by the recent dengue distribution maps produced by Samir Bhatt and colleagues (2013) [5]. Additionally, we combined species occurrence data from three vector databases (European Network for arthropod vector surveillance for human public health [VBORNET], Walter Reed Biosystematics Unit [WRBU] and Global Invasive Species Database) to provide the distribution of both vectors.

We aimed to identify countries/territories which report both chikungunya and dengue occurrence and to identify countries/territories that currently have endemic vectors but no reported local dengue or chikungunya transmission. Therefore, for mapping purposes, country level was used except for countries with a total area greater than 5,000,000 km^2^ for which province/region/state-level data were available.

## Results

### Reported and potential distribution of the viruses and mosquitoes

**Fig. 1:**
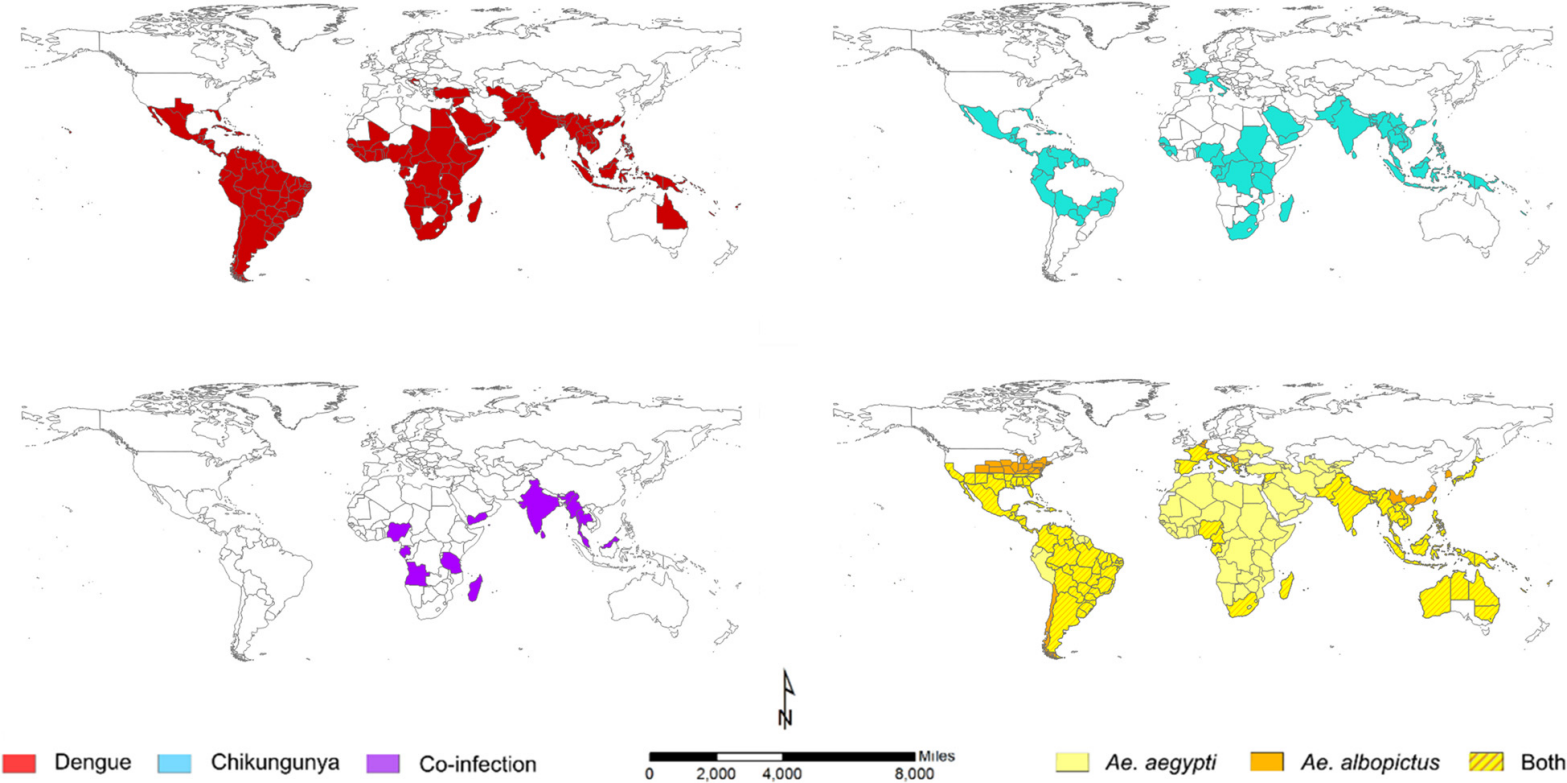
legend. The global distributions of endemic/epidemic dengue (*top left*) and chikungunya (*top right*) and reports of co-infection (*bottom left*) as well as the principal vectors of both arboviruses, *Aedes aegypti* and *Aedes albopictus* (*bottom right*)

Figure 1 shows the global distribution of chikungunya, dengue and co-infection as well as the principal vectors, *Ae. aegypti* and *Ae. albopictus.* A total of 154 (Fig. 1-*top left panel*) and 99 (Fig. 1-*top right panel*) countries/territories were found that reported endemic/epidemic dengue and chikungunya, respectively. Of the 98 countries/territories with reported local transmission for both chikungunya and dengue, only 13 have recorded co-infections (Fig. 1-*bottom left panel*). Fifty-six countries/territories are currently known to have endemic/epidemic dengue but are lacking evidence for ever having had local chikungunya transmission. One hundred and seventy-four countries/territories were found to have endemic *Ae. aegypti* populations and 88 countries/territories have *Ae. albopictus.* Only 68 countries/territories reported the presence of both vector species (Fig. 1-*bottom right panel*; Additional file 1: S3).

Regarding transmission, *Ae. aegypti* has historically been understood to be the vector of greatest public health significance for both DENV and CHIKV. We found no evidence for a substantial role of any alternative vector species prior to 2004. Although *Ae. aegypti* constituted the main vector species in Kenya during the major 2004 outbreak [51, 52], *Ae. albopictus* was the principal vector in succeeding epidemics in Gabon [30, 31], Madagascar [25] and La Reunion [53].

Chikungunya strains isolated from La Reunion were found to have a mutation at position 226 in the E1 envelope glycoprotein resulting in a significant increase in the infectivity of the virus to *Ae. albopictus* [54]. This vector species facilitated the 2007 autochthonous transmission of chikungunya in Italy following the virus’ introduction from a traveller returning from India [55], and may also be an important contributor to the recent chikungunya-dengue co-infections found in the Americas [47]. Vazeille et al. (2010) showed for the first time in an artificial infection experiment that the same *Ae. albopictus* mosquito could simultaneously be infected with CHIKV and DENV [56]. Subsequently, a naturally co-infected *Ae. albopictus* was discovered during the 2010 outbreak of both viruses in Gabon [30].

Among the studies that reported DENV/CHIKV co-infection only five studies conducted entomological surveys to assess the vector(s) involved in co-infection [20, 23, 25, 30, 31]. In the South-East Asian region, *Ae. aegypti* was the primary vector involved in the co-infection cases from 1964 in India [20] and 1970–72 in Myanmar [23]; whereas in the African region, *Ae. albopictus* was the responsible vector in Madagascar (2006) [25] and Gabon (2007-10) [30, 31]. Although, specific *Aedes spp* are known to be predominant in certain regions (e.g. *Ae. aegypti* in India), we cannot retrospectively ascertain which species was responsible for the spread of DENV/CHIKV in the remaining studies which did not report contemporaneous entomological surveys, due to the rapid changing distribution of both arbovirus vectors [57]. Caron et al. detected three *Aedes* spp. present in Gabon; however, only *Ae. albopictus* was found to be positive for both viruses, while *Ae. aegypti* was positive for CHIKV and *Ae. simpsoni* tested negative for DENV and CHIKV [30].

### Evidence of chikungunya-dengue co-infection

A total of 30 eligible studies were selected out of 129 identified in the combined search for chikungunya-dengue co-infection (S2). Reporting of chikungunya-dengue co-infection cases clearly depicts the spread of both viruses across countries/continents over time.

The first cases of dengue-chikungunya co-infection were reported in Thailand by Nimmannitya et al. who detected four co-infected cases among 150 patients diagnosed with either dengue or chikungunya (2.6 %) in 1962; three co-infected cases out of 144 infected patients (2.1 %) in 1963; and 12 co-infected cases out of 334 infected patients (3.6 %) in 1964 [18]. In 1964, co-infection cases were also reported in south India [19, 20] during a spate of chikungunya epidemics spanning 1963–1973 [21]. One hundred and ninety-five out of 372 patients presenting dengue-like illness were found to be chikungunya positive, one positive for DENV-1 and three positive for DENV-2 [19]. Among the patients with dengue-like illness, 2 % presented chikungunya-dengue co-infection [19, 20]. Recent phylogenetic analysis, based on the *Alphavirus* genus–specific NS4 gene, revealed the Indian CHIKV to be highly related (same within-clade cluster) to the Asian genotype responsible for the contemporaneous Thai outbreaks [22].

Active surveillance in the Children’s Hospital, Yangon General Hospital and the Defence Services Hospital in Myanmar identified 36 out of 539 (6.7 %) dengue and/or chikungunya positive patients to be co-infected in 1970; eight out of 129 (6.2 %) in 1971; and 11 out of 244 (4.5 %) in 1972 [23]. Following the studies reporting chikungunya-dengue co-infection in Thailand [18], India [19, 20] and Myanmar [23], no reports were found of chikungunya-dengue co-infection for more than 30 years despite sustained CHIKV and DENV endemicity in Africa and Asia.

In 2004, an outbreak of a new strain of chikungunya occurred in Lamu and then Mombasa on the Kenyan coast. Normally maintained in a sylvatic cycle in Kenya, this newly emergent strain from the Central/East African clade reached a very high attack rate of 75 % in the immunologically naïve local human populations [11]. Through international travel and transport of goods [24], it subsequently spread to islands of the Indian Ocean, India and South-East Asia. Consequently, in 2006 chikungunya-dengue co-infections were identified in Madagascar [25], Sri Lanka [26, 27], India [28] and Malaysia [29]. Between 2006 and 2012, numerous studies reported concurrent chikungunya-dengue infection during CHIKV or DENV outbreaks in Africa [30–32], South-East Asia [33–44], Eastern Mediterranean [45] and the Western Pacific region [46]. In December 2013, the first autochthonous case of chikungunya was reported in the Caribbean island of Saint Martin, and coincided with a dengue epidemic resulting in the first sixteen documented co-infected cases for the Americas [47]. Although, further cases of co-infection have not been reported in America, co-infection cases persist in Africa [48, 49] and South-East Asia [50]. A chronology of chikungunya-dengue co-infection reports by region/country, along with prevalence estimates between 1962 and 2015 is shown in Table 1.

**Table 1:** Characteristics of studies that reporting chikungunya-dengue co-infection

Location	Year	Study type	DENV+ and/or CHIKV+ cases	Co-infection cases	Co-infection prevalence (%)	Strains CHIKV/DENV	Vector	Laboratory method for CHIKV/DENV detection	Reference
*Africa Region*									
Angola	2014	Case report	NA	1	NA	CEA/4	NR	IgM ELISA/IgM ELISA + RT-PCR	[48]
Gabon	2007	Outbreak report	337	8	2.4	NR/2	*Ae. albopictus*	RT-PCR/RT-PCR	[31]
	2007	Surveillance	374	9	2.4	WA/2	*Ae. albopictus*	RT-PCR/RT-PCR	[30]
	2008	Surveillance	164	0	0	WA/2	*Ae. albopictus*	RT-PCR/RT-PCR	[30]
	2009	Surveillance	14	0	0	WA/2	*Ae. albopictus*	RT-PCR/RT-PCR	[30]
	2010	Surveillance	1400	28	2.0	WA/2	*Ae. albopictus*	RT-PCR/RT-PCR	[30]
Madagascar	2006	Cross-sectional	38	10	26.3	CEA/1	*Ae. albopictus*	IgM ELISA + RT-PCR/IgM ELISA + RT-PCR	[25]
Nigeria	2008	Cross-sectional	183	63	34.4	NR/NR	NR	PRNT/PRNT	[32]
	2014	Case report	NA	1	NA	NR/NR	NR	RT-PCR/RT-PCR	[50]
Tanzania	2013	Cross-sectional	93	4	4.3	NR/NR	NR	IgM ELISA/IgM ELISA + RT-PCR	[49]
*Region of the Americas*									
St. Martin	2013-14	Outbreak report	651	16	2.5	Asian/1,2,4	NR	IgM ELISA + RT-PCR/IgM ELISA + RT-PCR	[47]
*South-East Asian Region*									
India	1964	Case report	332	7	2.1	NR/2	NR	HI + Ig detection/HI + Ig detection	[19]
	1964	Cross-sectional	294	8	2.7	Asian/2	*Ae. aegypti*	HI + Ig detection/HI + Ig detection	[20]
	2006	Outbreak report	65	6	9.2	CEA/1,2,3,4	NR	RT-PCR/RT-PCR	[28]
	2007	Cross-sectional	387	8	2.1	NR/3,4	NR	RT-PCR/IgM ELISA + RT-PCR	[34]
	2008	Case report	NA	1	NA	NR/NR	NR	IgM IFA/IgM ELISA + IFA	[33]
	2009-10	Prospective	44	16	36.4	NR/NR	NR	IgM ELISA + RT-PCR/IgM ELISA	[42]
	2010	Cross-sectional	51	5	9.8	CEA/1	NR	RT-PCR/RT-PCR	[37]
	2010	Cross-sectional	73	4	5.5	NR/NR	NR	IgM ELISA/IgM ELISA	[43]
	2010	Cross-sectional	303	68	22.4	NR/2,3	NR	IgM ELISA/IgM ELISA	[38]
	2011	Cross-sectional	21	2	9.5	NR/NR	NR	IgM ELISA/IgM ELISA	[40]
	2011	Cross-sectional	68	9	13.2	CEA/1,2	NR	IgM ELISA + RT-PCR/IgM ELISA + RT-PCR	[41]
	2011-12	Cross-sectional	191	2	1.0	NR/NR	NR	IgM ELISA/IgM ELISA	[39]
	2012	Case report	NA	1	NA	NR/NR	NR	NR/NR	[44]
Myanmar	1970	Prospective	539	36	6.7	NR/NR	*Ae. aegypti*	HI + CF/HI + CF	[23]
	1971	Prospective	129	8	6.2	NR/NR	*Ae. aegypti*	HI + CF/HI + CF	[23]
	1972	Prospective	244	11	4.5	NR/NR	*Ae. aegypti*	HI + CF/HI + CF	[23]
	2010	Cross-sectional	60	7	11.7	CEA/NR	NR	IgM ELISA/IgM ELISA	[36]
Sri Lanka	2006	Case report	NA	1	NA	CEA/NR	NR	RT-PCR/RT-PCR	[26]
	2006-07	Prospective	44	3	6.8	CEA/NR	NR	IgM ELISA/IgM ELISA	[27]
Thailand	1962	Prospective	150	4	2.7	Asian/NR	NR	HI/HI + CF	[18]
	1963	Prospective	144	3	2.1	Asian/NR	NR	HI/HI + CF	[18]
	1964	Prospective	334	12	3.6	Asian/NR	NR	HI/HI + CF	[18]
	2009	Prospective	43	1	2.3	NR/NR	NR	RT-PCR/RT-PCR	[35]
*Eastern Mediterranean Region*									
Yemen	2012	Cross-sectional	165	14	8.5	NR/2	NR	IgM ELISA + RT-PCR/IgM ELISA + RT-PCR	[45]
*Western Pacific Region*									
Malaysia	2006	Case report	NA	2	NA	CEA/1	NR	RT-PCR/IgM ELISA	[29]
Singapore	2009	Case report	NA	1	NA	CEA/2	NR	RT-PCR/RT-PCR	[46]

*NA* not applicable, *NR* not reported, *CEA* Central/East African, *WA* West African, *HI* haemagglutination inhibition, *CF* complex fixation, *IFA* immunofluorescence assay, *PRNT* plaque reduction neutralization test

### Impact on diagnosis and clinical outcomes

**Fig. 2:**
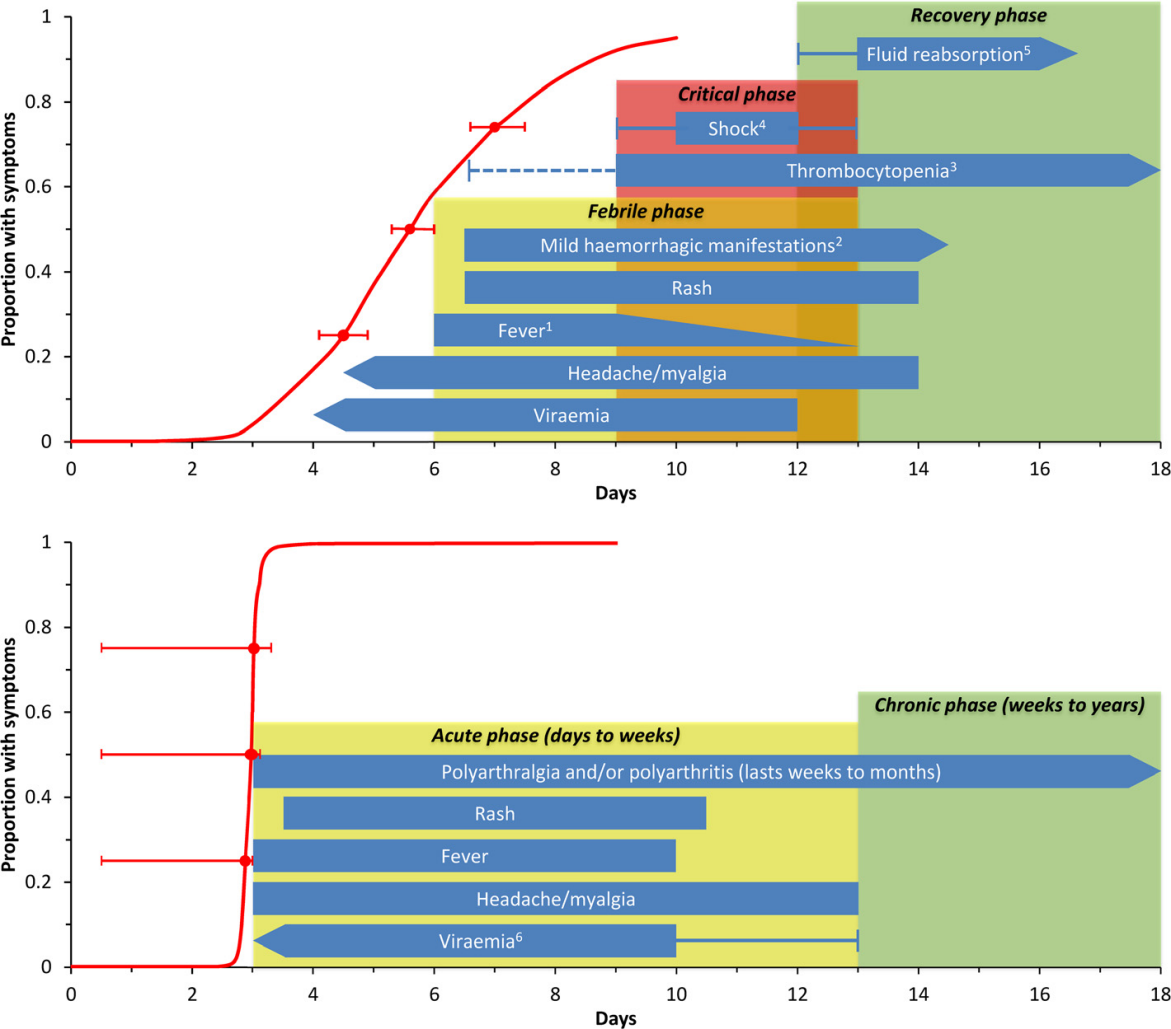
legend. Clinical symptoms typical of dengue (top) and chikungunya infections (bottom). The red line denotes the cumulative distributions (and 95 % CI at the 25^th^, 50^th^ and 75^th^ percentiles) for the incubation period of human infection (time between initial infection and symptoms onset) for both arboviruses as reported in a recent systematic review of Rudolph et al. [58]. *Dengue virus infection (top):* time course for the three phases of dengue infection (febrile, critical and recovery phase) are reproduced from WHO [92]. Boxes indicating typical signs/symptoms of dengue virus infection were reproduced from Whitehead *et al.* [91] unless otherwise indicated. Arrows indicate that signs/symptoms may occur earlier/later than illustrated (eg. headaches may occur earlier than 4.5 days post-infection). Notes: ^1^Onset of the critical phase usually coincides with defeverescence and is characterised by an increase in capillary permeability and significant plasma leakage lasting 1-2 days. Disease may resolve without entering the critical phase [93]. ^2^Mild haemorrhagic manifestations (mucosal bleeding/petechiae/bruising) may be observed from the febrile phase. Vaginal and intestinal bleeding may occur less commonly [92]. ^3^Platelet counts decline during the febrile phase (broken line), reaching lowest values at defeverescence. Thrombocytopenia, however, should not be used as an early indicator for development of severe disease (dengue haemorrhagic fever) as platelet counts in the early febrile phase do not vary markedly [93]. ^4^Hypovolemic shock typically lasts 1-2 days and can develop during late stages of the disease [91, 92]. ^5^During the recovery phase, reabsorption of extravascular compartment fluid occurs over 2-3 days [92]. *Chikungunya virus infection (bottom):* time course for the two phases of chikungunya infection (acute and chronic phase) and typical signs and symptoms are reproduced from Suhrbier et al*.* [90]. ^6^Viraemia typically lasts 5-7 days [90] and may precede the onset of symptoms. Viraemia in symptomatic patients typically peaks within the first three days [94] and has been reported to last for up to 11 days [95]. Viraemia has also been observed to persist in some patients for 2-3 days post- defervescence [95]

The progression of infection and symptoms for both chikungunya and dengue are shown in Fig. 2. Given that the symptoms associated with the acute phase of dengue mono-infection are often indistinguishable from those presented by patients with chikungunya infection [58], confirmatory laboratory diagnosis is required for appropriate treatment recommendation.

#### Detection of the viruses

The virus can be isolated during early stage infection by inoculating diagnostic samples into mosquitoes, mosquito cell lines, mammalian cell lines or the cerebra of suckling mice, and these were the methods generally used in the earlier studies [18–20, 23]. However, these methods are technically demanding, time consuming (up to a week), expensive and not very sensitive [61], and have consequently been superseded, in large part, by molecular methods. Most modern (post-2004) studies of co-infection have employed RT-PCR methods to detect viral nucleic acid because of improved sensitivity and rapidity (results are typically available within 1-2 days) [62, 63]. These methods were often complemented with immunoglobulin M and/or IgG detection or seroconversion using ELISA [25, 41, 45, 47, 64]. The indirect detection is easily performed but has sensitivities that are variable according to the stage of infection and the patient’s history of pathogen exposure [65].

While virus is only detectable within the first few days of symptoms onset (Fig. 2), antibodies take longer to develop and accumulate to detectable levels [65]. This transition in appropriate laboratory diagnostics according to temporality of infection is reflected in the dengue case investigation reporting procedure of the CDC, and discussed in a recent CDC expert commentary [66].

#### Clinical significance of co-infection

In terms of clinical outcome, only four studies have described the severity of dengue-chikungunya co-infection [28, 33, 38, 47]. Three studies indicated that neither symptoms nor clinical outcome were exacerbated by co-infection (relative to monotypic infection). Only Chahar et al. described a high rate of severe symptoms and poor clinical outcomes among co-infected patients [28]. Among the 6 co-infected patients, 2 developed DHF with central nervous system involvement and 1 ultimately died [28]. It is worth highlighting that the majority of dengue infections diagnosed during this latter study were secondary infections which may be associated with the observed high rates of severe disease without chikungunya involvement. Furthermore, no details were provided regarding the symptom severity of the dengue-infected but CHIKV-negative patients to allow comparison [28].

## Discussion

We are witnessing a rapid expansion in the geographical extent of chikungunya which mirrors that of dengue as described by Gubler in the 1990s [67]. This has come about partly through the increased opportunity for pathogen and vector spread that has resulted from globalisation [68], and the multifaceted effects on infectious diseases of a growing human population with resultant environmental changes [69]. Perhaps equally important, however, is the reporting bias that has obscured the public health impact of this pathogen, from its discovery until quite recently; CHIKV was first isolated in 1953 from the serum of a suspected dengue patient [70] and its conflation with dengue has persisted. Of the 30 studies eligible for inclusion in the current review, only one arose from an investigation of dengue cases, indicating a conspicuous absence of chikungunya diagnoses when dengue is suspected. Synthesising the available literature on chikungunya and dengue co-infection has revealed several limitations in our current understanding of the epidemiology of coinfection with both arboviruses and identified priorities for future research.

Similar to the global compendium of dengue [71], a consolidated, easily updateable and continuously maintained global database of chikungunya case notifications is needed and should be linked with reports of vector species detection. Subsequent to the 2006 chikungunya outbreak in French territory Le Reunion, several European countries (among them, France, Italy and Switzerland) have adopted a linked surveillance system for both arboviruses and vectors, with clear guidelines for curbing spread including educating inhabitants of outbreak foci on personal protection from mosquito bites, and rapid-response integrated vector management control campaigns [72]. Following France’s example, and, particularly in countries at the fringes of transmission and that have the facilities, both arboviruses must be nationally notifiable for this database to be useful in tracking the spread of disease with any fidelity. We note that this is easily implemented for countries that already have national notifiable databases for other diseases, and that are considered at high risk of incursion by these pathogens. One such example is Australia, which lists dengue as nationally notifiable but not chikungunya in all states and territories.

Improved cartographic refinement to a sub-national level is a logical next step that would build on the current exercise. While this was possible for some countries, data were not available to inform a global, sub-national level map. Differentiating endemic from epidemic regions for both chikungunya and dengue, and introducing an ordinal categorisation of disease level, such as has been developed for malaria [73], would enable tracking changes of the burden of disease and facilitate prioritisation of interventions. Enhanced geographical refinement and improved categorisation of at risk areas would not only enable focused targeting of surveillance and vector control, but also inform the denominator of co-infection prevalence.

In the current study we have identified a wide range of reported coinfection prevalence estimates (from 1.0–36.4 %); a key limitation with interpreting this finding is that it is set against a variable and dynamic background of monotypic infection prevalence. Furthermore, population standardised data is required to estimate the overall or by region DENV/CHIKV co-infection prevalence [74]; currently, it is not possible to compute a pooled estimate using the available data provided in the studies. Importantly, determining whether infection with one of the arboviruses enhances or attenuates host susceptibility to heterologous infection is not possible through indirect inference of relative prevalence levels; and this potential for ecological fallacy has been discussed fully in the context of more classically recognised mixed infections, for example the polyparasitism of soil-transmitted helminths [75]. The limited available information on infectivity of co-infected individuals provided by the 2012 Gabon study of Caron and colleagues suggests that co-infection reduces viral load relative to monotypic infection [30]. Determining how robust this result is across studies is important both immediately in terms of outbreak and control threshold estimation and in the longer term in the co-evolutionary context of these co-circulating pathogens.

Of related epidemiological significance is the determination of vector competence in virus-infected and superinfected mosquitoes [76, 77]. A recent review and modelling analysis by Christofferson et al. (2014) demonstrates the importance of considering the different combinations of pathogen-vector pairs at a finer resolution than serotype-genotype because of the variation in transmission potential found in even closely related strains [78]. Additionally, experiments suggest co-infection with multiple dengue serotypes may interfere with the vector’s ability to transmit virus [79]; whereas transmission enhancement has been demonstrated in the context of some other arboviruses [80]. Whether the chikungunya E1-226 V mutant that significantly increases chikungunya infectivity to *Ae. albopictus* also affects co-infected mosquitoes in their capacity as dengue vectors is unclear. Identifying any synergistic or antagonistic pathogen interactions within the vector constitutes an important, achievable future milestone in assessing the epidemiological consequences of chikungunya and dengue co-distribution.

The current study emphasises the likelihood of misdiagnosis of chikungunya infections among background dengue transmission (and vice versa). Critically, misdiagnosis not only hampers epidemiological understanding of both diseases but can profoundly affect the clinical picture of, and outcome for, infected patients. For example, misdiagnosis of dengue fever as chikungunya (or missing a dengue infection when coinciding with chikungunya) risks delaying or disrupting dengue-specific intensive supportive treatment [81] which can have a ten-fold impact on likelihood of progression from dengue fever to severe disease [82–85]. It also risks inappropriate prescription of arthralgia-alleviating nonsteroidal anti-inflammatory drugs (often employed in treating chikungunya patients) which could lead to severe bleeding in patients with thrombocytopenia or DHF [35]. The opposite and potentially more likely scenario in which chikungunya infection is misdiagnosed as dengue (or missed in a co-infected individual) masks the true geographical extent of CHIKV and population at risk of infection. It also obscures the likelihood of progression to severe disease in chikungunya patients: did the increased fatality rate reported post 2004 [11] result from a mutated CHIKV or was it simply easier to correctly attribute deaths from dengue-like illness due to increased awareness of chikungunya during the outbreak?

## Conclusions

In this study we provide evidence of widespread co-distribution and co-infection with dengue and chikungunya. Our results suggest that clear protocols are urgently needed for realistic and effective control procedures which a) include emergency responses that take advantage of the shared transmission route of these arboviruses, b) are tempered by local transmission settings and informed by linked pathogen-vector databases and c) capitalise upon modern modelling methods for informing both the biology of infection and transmission processes as well as the strategy and tactics of disease control. Quantitative methods have been capitalised upon to great effect in terms of geospatial statistical approaches for generating high-resolution global maps of dengue risk [5]; early warning systems of dengue outbreaks [86]; biologically detailed multi-serotype mathematical models of dengue spread and control [87, 88]; and combinations thereof [89]. The time is ripe to take advantage of these developments to accelerate corresponding developments for chikungunya as well as dengue-chikungunya co-distribution and co-infection, to facilitate a more holistic understanding of the rapidly evolving, global epidemiology of these arboviruses.

## Additional file

Additional file 1: Search strategy, study selection and country/territories with recorded transmission of dengue and chikungunya and presence of *Ae. aegypti* and *Ae. albopictus*. (DOCX 178 kb)

## Abbreviations

CDC: centers for disease and control prevention
CHIKV: Chikungunya virus
DENV: Dengue virus
DHF: Dengue haemorrhagic fever
ELISA: Enzyme-linked immunosorbent assay
Ig: Immunoglobulin
qPCR: Quantitative polymerase chain reaction
RT-PCR: Real time polymerase chain reaction
VBORNET: European Network for arthropod vector surveillance for human public health
WHO: World Health Organization
WRBU: Walter Reed Biosystematics Unit
