# Erratum to: Co-distribution and co-infection of chikungunya and dengue viruses

**DOI:** 10.1186/s12879-016-1519-x

**Published:** 2016-04-29

**Authors:** Luis Furuya-Kanamori, Shaohong Liang, Gabriel Milinovich, Ricardo J. Soares Magalhaes, Archie C. A. Clements, Wenbiao Hu, Patricia Brasil, Francesca D. Frentiu, Rebecca Dunning, Laith Yakob

**Affiliations:** Research School of Population Health, Australian National University, Acton, ACT 2601 Australia; Environmental Health Institute, National Environment Agency, Singapore, 138667 Singapore; School of Public Health and Social Work, Queensland University of Technology, Kelvin Grove, QLD 4059 Australia; School of Veterinary Science, University of Queensland, Gatton, QLD 4343 Australia; UQ Children’s Health Research Centre, University of Queensland, South Brisbane, QLD 4101 Australia; Instituto Nacional de Infectologia Evandro Chagas/Fiocruz, Rio de Janeiro, Brazil; School of Biomedical Sciences and Institute for Health and Biomedical Innovation, Queensland University of Technology, Kelvin Grove, QLD 4059 Australia; Formerly School of Biomedical Sciences, University of Queensland, St Lucia, QLD 4072 Australia; Department of Disease Control, London School of Hygiene and Tropical Medicine, London, WC1E 7HT UK

## Erratum

After publication of the original article [1], it came to the authors’ attention that there were errors within Fig. 1 and Additional file 1.

**Fig. 1:**
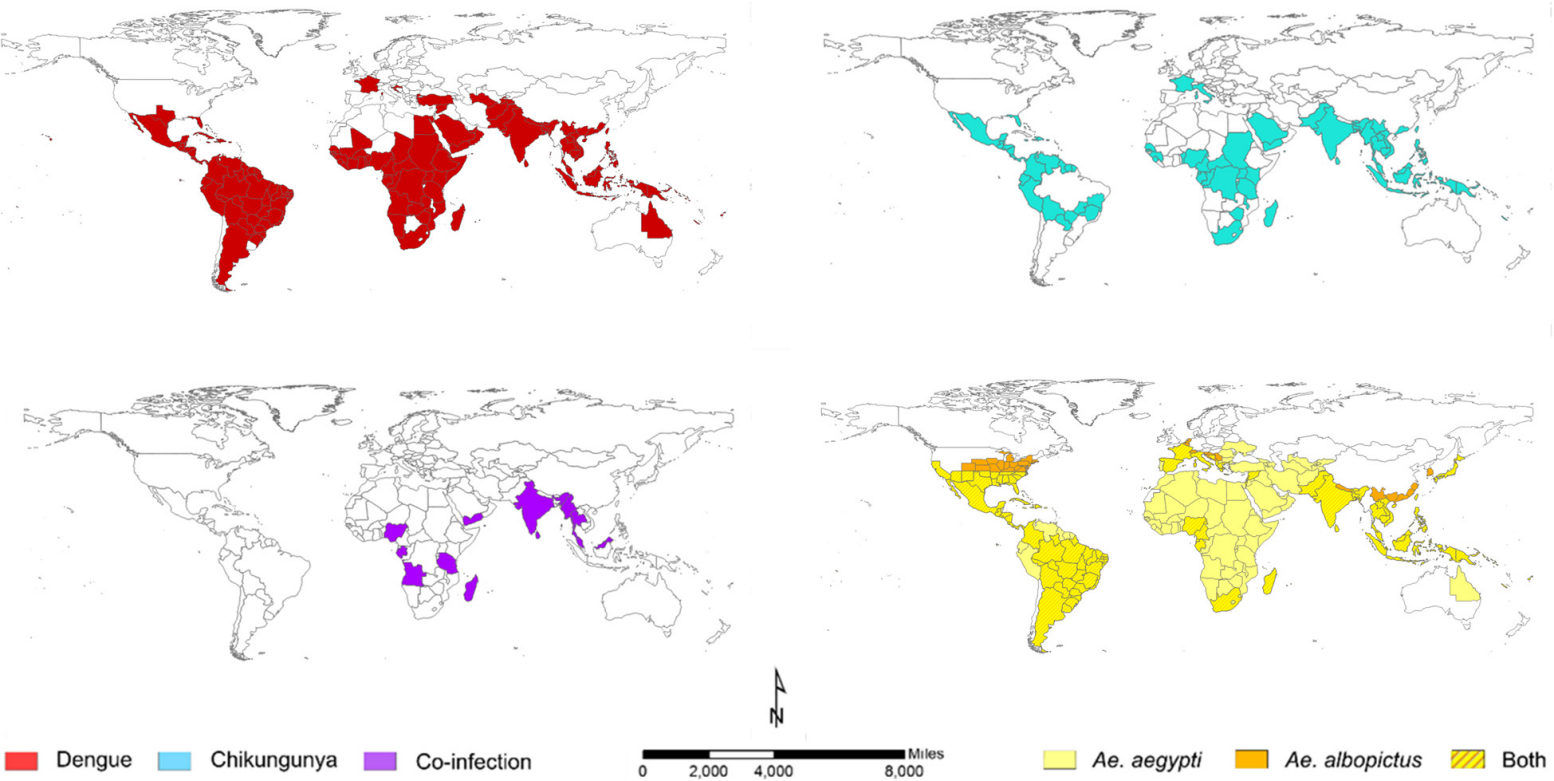
The global distributions of endemic/epidemic dengue (*top left*) and chikungunya (*top right*) and reports of co-infection (*bottom left*) as well as the principal vectors of both arboviruses, *Aedes aegypti* and *Aedes albopictus* (*bottom right*)

The original article has been updated to include the correct versions of Fig. 1 and Additional file 1 have been updated, which also appear in this erratum.

## Additional file

Additional file 1: Search strategy, study selection and country/territories with recorded transmission of dengue and chikungunya and presence of *Ae. aegypti* and *Ae. albopictus*. (DOCX 26 kb)
