# Supplementary material for: Erratum to: Co-distribution and co-infection of chikungunya and dengue viruses
Source: BMC Infect Dis. 2016 Apr 29;16:188. doi: 10.1186/s12879-016-1519-x (PMC4851825; doi:10.1186/s12879-016-1519-x)
Supplement: Additional file 1: — Search strategy, study selection and country/territories with recorded transmission of dengue and chikungunya and presence of Ae. aegypti and Ae. albopictus. (DOCX 26 kb) [file 12879_2016_1519_MOESM1_ESM.docx]

| COUNTRY | D+ | C+ | V+ |
| --- | --- | --- | --- |
| Afghanistan | 1 |  | 1 |
| Albania |  |  | 3 |
| Algeria |  |  | 1 |
| American Samoa | 1 | 1 | 3 |
| Angola | 1 |  | 1 |
| Anguilla | 1 | 1 | 1 |
| Antigua and Barbuda | 1 | 1 | 1 |
| Argentina | 1 |  | 3 |
| Armenia |  |  | 1 |
| Aruba | 1 | 1 | 1 |
| Australia | 1 |  | 1 |
| Azerbaijan |  |  | 1 |
| Azores |  |  | 1 |
| Bahamas | 1 | 1 | 1 |
| Bahrain |  |  | 1 |
| Bangladesh | 1 | 1 | 3 |
| Barbados | 1 | 1 | 3 |
| Belgium |  |  | 2 |
| Belize | 1 | 1 | 1 |
| Benin | 1 | 1 | 1 |
| Bermuda | 1 |  | 1 |
| Bhutan | 1 | 1 | 3 |
| Bolivia | 1 | 1 | 3 |
| Borneo |  |  | 2 |
| Bosnia and Herzegovina |  |  | 3 |
| Botswana |  |  | 1 |
| Brazil | 1 | 1 | 3 |
| British Indian Ocean Territory |  |  | 2 |
| COUNTRY | D+ | C+ | V+ |
| British Virgin Islands | 1 | 1 | 1 |
| Brunei | 1 |  | 3 |
| Bulgaria |  |  | 1 |
| Burkina Faso | 1 |  | 1 |
| Burundi |  | 1 | 1 |
| Cambodia | 1 | 1 | 3 |
| Cameroon | 1 | 1 | 3 |
| Cape Verde | 1 |  | 3 |
| Cayman Islands | 1 | 1 | 3 |
| Central African Republic | 1 | 1 | 1 |
| Chad | 1 |  | 1 |
| China | 1 | 1 | 3 |
| Colombia | 1 | 1 | 3 |
| Comoros | 1 | 1 | 2 |
| Congo |  | 1 | 1 |
| Cook Islands | 1 | 1 | 3 |
| Costa Rica | 1 | 1 | 3 |
| Cote d'Ivoire | 1 |  | 1 |
| Croatia | 1 |  | 2 |
| Cuba | 1 |  | 3 |
| Curacao | 1 | 1 | 1 |
| Democratic Republic of Congo | 1 | 1 | 1 |
| Djibouti | 1 |  | 1 |
| Dominica | 1 | 1 | 1 |
| Dominican Republic | 1 | 1 | 3 |
| East Timor | 1 | 1 | 1 |
| Ecuador | 1 | 1 | 1 |
| Egypt | 1 |  | 1 |
| COUNTRY | D+ | C+ | V+ |
| El Salvador | 1 | 1 | 3 |
| Equatorial Guinea | 1 | 1 | 3 |
| Eritrea | 1 |  | 1 |
| Ethiopia | 1 |  | 1 |
| Fiji | 1 |  | 3 |
| Federated States of Micronesia | 1 | 1 | 1 |
| France |  | 1 | 3 |
| French Guiana | 1 | 1 | 1 |
| French Polynesia | 1 |  | 2 |
| Gabon | 1 | 1 | 3 |
| Gambia | 1 |  | 1 |
| Georgia |  |  | 1 |
| Ghana | 1 |  | 1 |
| Greece |  |  | 3 |
| Grenada | 1 | 1 | 1 |
| Guadeloupe | 1 | 1 | 1 |
| Guatemala | 1 | 1 | 3 |
| Guinea | 1 | 1 | 1 |
| Guinea-Bissau | 1 |  | 1 |
| Guyana | 1 | 1 | 1 |
| Haiti | 1 | 1 | 1 |
| Honduras | 1 | 1 | 3 |
| Hong Kong | 1 |  | 2 |
| India | 1 | 1 | 3 |
| Indonesia | 1 | 1 | 3 |
| Iran |  |  | 1 |
| Iraq |  |  | 1 |
| Israel |  |  | 3 |
| COUNTRY | D+ | C+ | V+ |
| Italy |  | 1 | 3 |
| Ivory Coast |  |  | 1 |
| Jamaica | 1 | 1 | 1 |
| Japan |  |  | 3 |
| Jordan |  |  | 1 |
| Kenya | 1 | 1 | 1 |
| Kiribati | 1 | 1 | 1 |
| Kuwait |  |  | 1 |
| Kyrgyzstan |  |  | 1 |
| Laos | 1 | 1 | 3 |
| Lebanon |  |  | 3 |
| Lesotho |  |  | 1 |
| Liberia | 1 |  | 1 |
| Libya |  |  | 1 |
| Macao | 1 |  | 1 |
| Macedonia |  |  | 1 |
| Madagascar | 1 | 1 | 3 |
| Malawi |  | 1 | 1 |
| Malaysia | 1 | 1 | 3 |
| Maldives | 1 | 1 | 2 |
| Mali | 1 |  | 1 |
| Marshall Islands | 1 |  | 1 |
| Martinique | 1 | 1 | 1 |
| Mauritania |  |  | 1 |
| Mauritius | 1 | 1 | 2 |
| Mayotte | 1 | 1 | 3 |
| Mexico | 1 | 1 | 3 |
| Montenegro |  |  | 2 |
| COUNTRY | D+ | C+ | V+ |
| Montserrat | 1 | 1 | 1 |
| Morocco |  |  | 1 |
| Mozambique | 1 |  | 1 |
| Myanmar | 1 | 1 | 3 |
| Namibia | 1 |  | 1 |
| Nauru | 1 |  | 1 |
| Nepal | 1 |  | 2 |
| Netherlands |  |  | 2 |
| Netherlands Antilles | 1 |  | 1 |
| New Caledonia | 1 | 1 | 3 |
| Nicaragua | 1 | 1 | 3 |
| Niger |  |  | 1 |
| Nigeria | 1 | 1 | 3 |
| Niue | 1 |  | 1 |
| Northern Mariana Islands | 1 |  | 2 |
| Oman | 1 |  | 1 |
| Pakistan | 1 | 1 | 3 |
| Palau | 1 |  | 2 |
| Panama | 1 | 1 | 3 |
| Papua New Guinea | 1 | 1 | 3 |
| Paraguay | 1 | 1 | 3 |
| Peru | 1 | 1 | 1 |
| Philippines | 1 | 1 | 3 |
| Portugal |  |  | 1 |
| Puerto Rico | 1 | 1 | 1 |
| Qatar |  |  | 1 |
| Reunion | 1 | 1 | 3 |
| Romania |  |  | 1 |
| COUNTRY | D+ | C+ | V+ |
| Rwanda | 1 |  | 1 |
| Saint Kitts and Nevis | 1 | 1 | 1 |
| Saint Lucia | 1 | 1 | 1 |
| Saint Vincent & Grenadines | 1 | 1 | 1 |
| Samoa | 1 | 1 | 3 |
| Sao Tome and Principe | 1 |  | 1 |
| Saudi Arabia | 1 | 1 | 1 |
| Senegal | 1 | 1 | 1 |
| Serbia |  |  | 2 |
| Seychelles | 1 | 1 | 2 |
| Sierra Leone | 1 | 1 | 1 |
| Singapore | 1 | 1 | 3 |
| Slovenia |  |  | 2 |
| Solomon islands | 1 |  | 3 |
| Somalia | 1 |  | 1 |
| South Africa | 1 | 1 | 3 |
| South Korea |  |  | 2 |
| Spain |  |  | 3 |
| Sri Lanka | 1 | 1 | 3 |
| Sudan | 1 | 1 | 1 |
| Suriname | 1 | 1 | 1 |
| Swaziland |  |  | 1 |
| Switzerland |  |  | 2 |
| Syria | 1 |  | 3 |
| Taiwan | 1 | 1 | 3 |
| Tajikistan | 1 |  | 1 |
| Tanzania | 1 | 1 | 1 |
| Thailand | 1 | 1 | 3 |
| COUNTRY | D+ | C+ | V+ |
| Togo | 1 |  | 1 |
| Tokelau | 1 | 1 | 1 |
| Tonga | 1 | 1 | 3 |
| Trinidad and Tobago | 1 | 1 | 3 |
| Tunisia |  |  | 1 |
| Turkey | 1 |  | 1 |
| Turkmenistan | 1 |  | 1 |
| Turks and Caicos Islands | 1 | 1 | 1 |
| Tuvalu | 1 |  | 3 |
| Uganda | 1 | 1 | 1 |
| Ukraine |  |  | 1 |
| United Arab Emirates | 1 |  | 1 |
| United States | 1 | 1 | 3 |
| Uruguay |  |  | 3 |
| Uzbekistan |  |  | 1 |
| Vanuatu | 1 |  | 3 |
| Venezuela | 1 | 1 | 1 |
| Vietnam | 1 | 1 | 3 |
| Virgin Islands, U.S. | 1 | 1 | 1 |
| Wallis and Futuna | 1 |  | 1 |
| Yemen | 1 | 1 | 1 |
| Zambia | 1 |  | 1 |
| Zimbabwe | 1 | 1 | 1 |
